# Effector and regulatory B-cell imbalance in systemic sclerosis: cooperation or competition?

**DOI:** 10.1007/s10067-024-07086-0

**Published:** 2024-07-30

**Authors:** Mengguo Liu

**Affiliations:** Department of Dermatology, Huashan Hospital, Fudan University, the 12Th Urumqi Road, Shanghai, 200040 China

**Keywords:** Systemic sclerosis, Effector B cells, Regulatory B cells, Autoimmunity

## Abstract

B cells play a central role in the pathogenesis of systemic sclerosis (SSc). Most B-cell studies have focused on their pathological role as antibody producers. However, in addition to immunoglobulin secretion, these cells have a wide range of functions in the immune response, including antigen presentation to T cells and cytokine production. Importantly, not all B-cell subsets promote the immune response. Regulatory B cells (Bregs) attenuate inflammation and contribute to the maintenance of immune tolerance. However, effector B cells (Beffs) positively modulate the immune response through the production of various cytokines. In SSc, Bregs are insufficient and/or dysfunctional. B-cell-targeting biologics have been trialled with promising results in the treatment of SSc. These therapies can affect Bregs or Beffs, which can potentially limit their long-term efficacy. Future strategies might involve the modulation of effector B cells in combination with the stimulation of regulatory subsets. Additionally, the monitoring of individual B-cell subsets in patients may lead to the discovery of novel biomarkers that could help predict disease relapse or progression. The purpose of this review is to summarize the relevant literatures and explain how Bregs and Beffs jointly participate in the pathogenesis of SSc.

## Introduction

Systemic sclerosis (SSc, also known as scleroderma) is a connective tissue disease that is characterized by excessive fibrosis of the skin and various internal organs and has an autoimmune aetiology [1]. The pathogenesis of SSc is unclear. More than 90% of SSc patients are positive for autoantibodies, such as anti–DNA topoisomerase I, anticentromere, and anti–RNA polymerase antibody (Ab) [2]. Increasing evidence shows that B cells are involved in SSc. One study demonstrated that SSc patients displayed distinct abnormalities in blood B-cell compartments; these abnormalities were characterized by expanded naïve B cells and activated memory B cells [3]. The sensitive balance between effector B cells (Beffs) and regulatory B cells (Bregs) is disrupted, the number of Bregs is reduced, and Bregs are functionally impaired [4]. Furthermore, studies have shown that interleukin-6 (IL-6) derived from effector B cells can promote fibrosis. However, regulatory B-cell-derived interleukin-10 (IL-10) has anti-inflammatory effects [5].

B cells promote the pathogenesis of SSc through diverse mechanisms, most notably via the production of antibodies, but also through antigen presentation, which leads to T cell activation and the secretion of various cytokines [6]. The crucial role of B cells in SSc is highlighted by the success of therapeutic B-cell depletion strategies [7, 8]. Research in preclinical models, together with insights from clinical studies of B-cell depletion therapy, has improved our understanding of the contribution of B cells to the aetiology and pathogenesis of SSc. B cells are important for Ab production, antigen presentation, and cytokine production [9]. In particular, cytokine-producing B cells play critical roles in multiple aspects of immunity. There are two opposing B-cell subsets: Bregs and Beffs [10]. Bregs are capable of modulating immune responses and contributing to immune tolerance [11]. B-cell depletion therapies target Beffs as well as Bregs; however, to date, no specific method has been developed to regulate Beffs without affecting Bregs. In addition, studies of B-cell subsets and their plasticity in patients with scleroderma are lacking. Understanding B-cell development and differentiation, as well as phenotypic and functional heterogeneity, will make the treatment of SSc by targeting B cells a possibility.

## B-cell differentiation and development

The development of B cells begins in the foetal liver and continues in the bone marrow, where multiple checkpoints ensure that B cells released to the periphery express functional B-cell receptors (BCRs) and are not specific for their own antigens [12]. The autoreactivity of BCR can be detected in the immature B-cell stage [13]. Early bone marrow transitional B lymphocytes are also called transitional B cells. These immature B cells express immunoglobulin M (IgM) and immunoglobulin D (IgD) on their surface, which are usually characterized by a CD24^hi^ CD38^hi^ phenotype and a lack of the memory B-cell marker CD27 [14]. Immature B cells develop in the periphery and mature into antigen-naïve CD24^int^ CD38^int^ CD27^−^ B cells [15]. Under antigen-specific stimulation, B cells can differentiate into plasmablasts, plasma cells and memory B cells [16]. Plasma cells are long-lived, terminally differentiated and antibody-producing cells, and plasmablasts are their short-lived proliferative counterparts [17]. These two cell types lack the expression of the pan-B-cell marker CD20 and are collectively referred to as antibody-secreting cells [18]. In the case of reinfection, plasma cells constantly produce antibodies in the bone marrow to provide immediate protection to the host. Memory B cells can be rapidly induced to differentiate into antibody-producing plasma cells through interactions with homologous T cells. This memory response is usually stronger than that of primary B cells to antigens, and these cells produce immunoglobulins with high affinity [19].

## The definition of Beffs and Bregs: the lack of a specific phenotype

There are no phenotypic markers or transcription factors that can be used to specifically identify either Beffs or Bregs. However, Beffs and Bregs are defined by the expression of proinflammatory or anti-inflammatory cytokines. Beffs can positively modulate the immune response through the production of various cytokines [20]. For example, lymphotoxin-producing Beffs are essential for the ontogenesis, homeostasis, and the activation of secondary lymphoid organs, as well as the development of tertiary lymphoid tissues at ectopic sites. Other Beffs have been shown to modulate the development of effector and memory CD4^+^ T-cell responses via the production of cytokines such as IL-6, interferon-γ (IFN-γ), tumour necrosis factor-α (TNF-α) and granulocyte macrophage colony stimulating factor (GM-CSF) 20]. In contrast, IL-10-producing Bregs are now recognized as negative regulators of the immune system, inflammation, and autoimmunity based on studies with human subjects and mouse models of autoimmune diseases such as rheumatoid arthritis, systemic lupus erythematosus (SLE), and multiple sclerosis (MS) [21–23] . The phenotype of mouse splenic Bregs is derived from two different B-cell subsets: the marginal zone (MZ) and B1 B cells [24]. Furthermore, the CD9^+^ B-cell subset is enriched in IL-10-producing Bregs since this subset includes both the MZ and B1 B-cell subsets [25]. Therefore, a protocol that selectively depletes Beffs while sparing Bregs would be a potent therapy for autoimmune diseases.

## Effector B cells in SSc

Beffs promote immune responses through the production of various proinflammatory cytokines [26]. In particular, IL-6- or GM-CSF-producing Beffs have emerged as important in the treatment of SSc [27, 28].

B cells are the main source of IL-6 [29]. IL-6 can induce naïve CD4^+^ T cells to differentiate into T follicular fluid (Tfh) and Th17 cells and induce B cells to differentiate into plasma cells [30, 31]. In addition, IL-6 also plays an important role in SSc. In a bleomycin-induced scleroderma model, IL-6 deficiency in B cells can reduce skin fibrosis, while IL-10 deficiency in B cells can increase skin fibrosis [32, 33]. IL-6 can also promote the secretion of collagen by fibroblasts [34]. Importantly, B-cell activating factor (BAFF) has been shown to increase IL-6^+^ Beffs [35]. In addition, BAFF antagonists reduce skin and pulmonary fibrosis in scleroderma models by reducing IL-6^+^ Beffs rather than IL-10^+^ Bregs [33]. In humans, B cells have been shown to secrete high levels of IL-6 in SSc patients [36]. Therefore, IL-6^+^ Beffs promote the pathogenesis of SSc. In an SSc mouse model, IL-6 played a key role in tissue fibrosis and autoimmunity [37]. Administration of an anti-IL-6 receptor Ab to mice reduced skin fibrosis in a mouse model of scleroderma. Studies have shown that skin and lung fibrosis in a scleroderma mouse model was attenuated in B-cell-specific IL-6-deficient mice.

GM-CSF was originally considered a haematopoietic growth factor that can induce the proliferation and differentiation of granulocytes and macrophages. However, GM-CSF also has a proinflammatory effect and plays a key role in autoimmune diseases [38, 39]. For example, GM-CSF is involved in the differentiation of fibroblasts into myofibroblasts, which may promote fibrosis in the pathogenesis of SSc [40]. A recent study showed that the Th2 cytokine IL-4 induces effector B cell production of GM-CSF (GM-Beffs) in humans [41]. GM-Beffs were enriched within CD20^+^CD30^+^CD38^−/low^ cells, a population distinct from plasmablasts, suggesting that GM-Beffs exert antibody-independent functions [28]. The level of CD30^+^GM-Beffs were significantly greater in SSc patients than in healthy controls. A subpopulation of SSc patients with diffuse-type and concomitant interstitial lung disease exhibited high numbers of GM-Beffs. In summary, these findings suggest that human GM-Beffs are enriched in the CD30^+^ B-cell subset and play a role in the pathogenesis of SSc [28].

## Regulatory B cells in SSc

Negative regulation of the immune response by B-cell subsets, called Bregs, is considered an important part of the immune system. Bregs play a crucial role in SSc. The production of IL-10 by Bregs is mainly related to the immune response [42]. IL-10 is an anti-inflammatory cytokine that negatively regulates the production of proinflammatory cytokines in T cells and the function of antigen-presenting cells (APCs), as well as dendritic cells and macrophages [43]. IL-10 is produced by a variety of immune cells, such as T cells, macrophages and B cells. IL-10-producing Bregs (IL-10^+^ Bregs) have been considered the main subgroup of cells that regulate the immune response because of their important functions. Mizoguchi et al. used the term “regulatory B cells” for the first time [44].

In SSc, IL-10^+^ Bregs have an inhibitory effect on pathogenesis [45]. Previous studies have shown that donor-derived IL-10^+^ Bregs are important for the suppression of SSc in the sclerodermatous chronic graft-versus-host disease (Scl-cGVHD) model [46]. The absence of IL-10^+^ Bregs induced severe Scl-cGVHD. In humans, the level of IL-10^+^ Bregs in patients with SSc is reduced, and a reduced level of IL-10^+^ Bregs is associated with interstitial lung disease [47, 48]. Moreover, the number of IL-10^+^ Bregs was negatively correlated with the extent of SSc in patients. In a follow-up study, compared with those before treatment, IL-10^+^ Breg levels in patients with SSc were found to be significantly increased after treatment, accompanied by decreased disease activity [49]. These results suggest that a decrease in the number of IL-10^+^ Bregs contributes to the development of SSc.

## Potential relevance of Beffs and Bregs in SSc

Overall, different approaches have been tested to address the hyperactivation of B cells in SSc. Currently, some success has been achieved in targeting B cells for SSc therapy. B-cell depletion therapy with rituximab, a CD20 Ab that depletes human pan-B cells, has shown beneficial effects on skin fibrosis, pulmonary function and pulmonary hypertension in patients with SSc [7, 50, 51]. Furthermore, in a phase 2 study and a phase 3 study, the IL-6-receptor-α inhibitor tocilizumab failed to reduce skin thickening, but tended to improve the modified Rodnan Skin Score and pulmonary function [52, 53]. The clinical improvement in both trials might have been limited by the lack of specificity, as depletion of all B-cell subsets eliminated the protective effects of Bregs.

IL-6 is a pleiotropic cytokine whose activity stimulates the proliferation and differentiation of B and T lymphocytes, increased antibody production, activates T cells, stimulates the differentiation of haematopoietic precursor cells, affects the proliferation of nonlymphoid cells, and activates the acute phase protein response [54]. IL-6 plays a special role in the development of SSc, both in vascular damage and in the development of fibrosis. In the early stages, IL-6 participates in vascular endothelial activation and apoptosis [55]. Moreover, IL-6 plays an important role in the development of fibrotic changes by mediating the transformation of fibroblasts into myofibroblasts. All of these conditions are associated with disabling clinical manifestations, such as skin thickening, pulmonary fibrosis, pulmonary arterial hypertension (PAH), heart failure, and dysphagia. IL-6 induced a concentration-dependent increase in collagen and glycosaminoglycan production by human dermal fibroblasts in vitro [56]. Many reports have shown that the production of IL-6 in fibroblasts from SSc-affected skin is increased compared with that in normal fibroblasts [57]. In addition, blocking the IL-6 response with an anti-IL-6 antibody resulted in a significant reduction in type I procollagen in cultured SSc fibroblasts [58]. A previous study also revealed that the level of serum IL-6 was closely related to skin thickening, which confirmed the important role of IL-6 in the development of SSc skin sclerosis [59, 60].

IL-10 is an anti-inflammatory cytokine that can inhibit the synthesis of proinflammatory cytokines in monocytes or macrophages [61]. IL-10 can stimulate B cells to produce IgG, IgA and IgM. In vitro experiments showed that IL-10 could downregulate the expression of the type I collagen gene and increase the expression of the collagenase gene [62]. Another study revealed a negative correlation between IL-10^+^ B cells and IL-17^+^ T cells in SSc patients who were positive for the anti-topo I antibody and the anticentromere Ab [49]. The number of IL-10^+^B cells in SSc patients is very low, especially in those with SSc-related interstitial lung disease (ILD); furthermore, the number of IL-10^+^B cells is negatively correlated with the number of IL-17A-producing T cells, suggesting that IL-10 plays an important negative regulatory role in the pathogenesis of SSc. IL-10 was found to inhibit helper T-cell 1 and 17 (Th1 and Th17) immune responses [63]. However, Th17 cells play an important role in the pathogenesis of SSc. In a mouse fibroblast line, IL-17A increased the expression of TGF-β, connective tissue growth factor (CTGF) and collagen but increased skin and lung inflammation and fibrosis in a mouse bleomycin (BLM) model of SSc [64, 65]. The role of Th17 cells in human SSc may not involve direct fibrosis but indirect promotion of inflammation and vasculopathy [66, 67]. Elevated levels of IL-17 were detected in the peripheral blood, skin and lung lymphocytes of patients with SSc [68, 69]. In human SSc, IL-17 increases fibroblast proliferation and collagen production and induces fibroblasts to produce IL-6, IL-8 and adhesion molecules [70, 71]. IL-17A also induces human endothelial cells to produce IL-6 and adhesions [72, 73]. Furthermore, IL-17A promotes the proliferation, migration, collagen synthesis and secretion of SSc patient-derived dermal vascular smooth muscle cells, resulting in intimal thickening and aggravated vasculopathy and fibrosis [74–76].

**Fig. 1:**
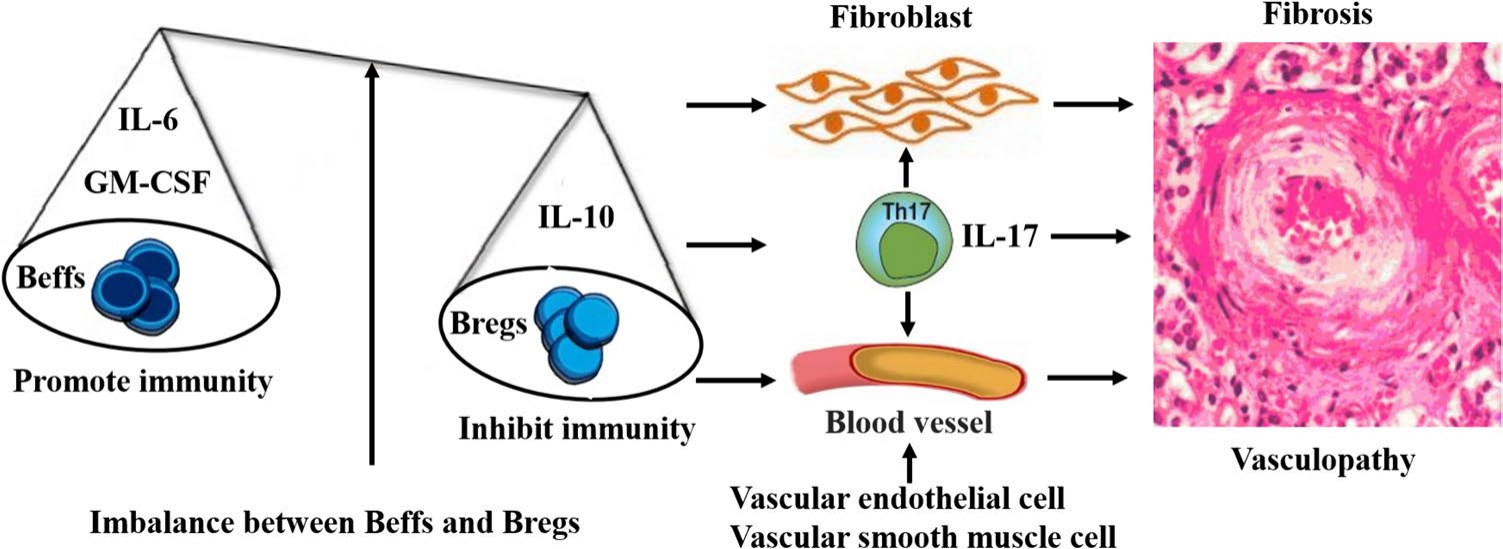
Disequilibrium between Beffs and Bregs in the pathogenesis of systemic sclerosis. There may be an imbalance between IL-6^+^ Beffs and IL-10^+^ Bregs in the pathogenesis of systemic sclerosis, as directly acting on fibroblasts and vascular endothelial cells or indirectly inducing Th17 cells to play a pathogenic role could be involved in inflammation, abnormal immunity, vasculopathy and fibrosis

Consequently, we speculate that there may be an imbalance between IL-6^+^ Beffs and IL-10^+^ Bregs in the pathogenesis of systemic sclerosis and that directly acting on fibroblasts and vascular endothelial cells, or indirectly inducing Th17 cells to play a pathogenic role, could be involved in inflammation, abnormal immunity, vasculopathy and fibrosis (Fig. 1).

## Conclusion

B cells both positively and negatively regulate the pathogenesis of SSc via cytokine production. However, identifying the definitive markers and specific transcription factors of Bregs and Beffs is necessary for the development of new B-cell-targeted therapeutic strategies. Nevertheless, Bregs and Beffs are critically important components of the immune system. B cells are the key factor in the pathogenesis of SSc. Therefore, in the future, increasing the number of Bregs or reducing the number of Beffs may be one of the best strategies to treat refractory autoimmune diseases such as SSc.

## Data Availability

Data availability is not applicable to this article as no new data were created or analyzed in this study.

## Abbreviations

Ab: Antibody
APC: Antigen presenting cells
BAFF: B-cell activating factor
BCRs: B-cell receptors
Beffs: Effector B cells
BLM: Bleomycin
Bregs: Regulatory B cells
cGVHD: Chronic graftversus-host disease
CTGF: Connective tissue growth factor
DC: Dendritic cells
GM-CSF: Granulocyte macrophage colony stimulating factor
IFN-γ: Interferon-γ
IgD: Immunoglobulin D
IgM: Immunoglobulin M
IL-6: qinterleukin-6
IL-10: Interleukin-10
ILD: Interstitial lung disease
MS: Multiple sclerosis
MZ: Marginal zone
SLE: Systemic lupus erythematosus
SSc: Systemic sclerosis
Tfh: T follicular fluid
Th1: Helper T-cell 1
Th17: Helper T-cell 17
TNF-α: Tumour necrosis factor-α
